# TEsorter: An accurate and fast method to classify LTR-retrotransposons in plant genomes

**DOI:** 10.1093/hr/uhac017

**Published:** 2022-04-11

**Authors:** Ren-Gang Zhang, Guang-Yuan Li, Xiao-Ling Wang, Jacques Dainat, Zhao-Xuan Wang, Shujun Ou, Yongpeng Ma

**Affiliations:** 1 Yunnan Key Laboratory for Integrative Conservation of Plant Species with Extremely Small Populations, Kunming Institute of Botany, Chinese Academy of Sciences, Kunming 650201, China; 2 Department of Bioinformatics, Ori (Shandong) Gene Science and Technology Co., Ltd., Weifang， Shandong 261322, China; 3 BGI-Shenzhen, Shenzhen 518083, China; 4 Department of Medical Biochemistry and Microbiology, National Bioinformatics Infrastructure Sweden, Science for Life Laboratory, Uppsala University, Uppsala, Sweden; 5 Shijiazhuang People’s Medical College, Shijiazhuang, Hebei 050091, China; 6 Department of Ecology, Evolution, and Organismal Biology (EEOB), Iowa State University, Ames, IA 50010, USA

Dear Editor,

Transposable elements (TEs) constitute the largest portion of repetitive sequences in many eukaryotic genomes, with long terminal repeat retrotransposons (LTR-RTs) being predominant in plant genomes. Various tools have been developed for the identification and classification of TEs, including RepeatModeler [1], REPET [2], LTR_retriever (https://github.com/oushujun/LTR_retriever), and TERL (https://github.com/muriloHoracio/TERL). To our knowledge, most existing software can only classify TEs to the superfamily level, in particular the LTR-RT *Copia* and *Gyspy* superfamilies in plants, leaving a significant knowledge gap. Moreover, although approaches for automated classification of LTR lineages using amino acid hidden Markov models (HMMs) do exist, these are typically comprised of collections of scripts that are not curated or specifically designed to be user-friendly.

Previous studies have proposed classifications of LTR-RTs at the clade level [3]. Neumann *et al*. [4] classified the *Copia* superfamily into the *Ale*, *Alesia*, *Angela*, *Bianca*, *Bryco*, *Lyco*, *Gymco* I–IV, *Ikeros*, *Ivana*, *Osser*, *SIRE*, *TAR*, and *Tork* clades and the *Gypsy* superfamily into the *CRM*, *Chlamyvir*, *Galadriel*, *Tcn1*, *Reina*, *Tekay*, *Athila*, *Tat* I–III, *Ogre*, *Retand*, *Phygy*, and *Selgy* clades. These studies provide protein domain databases for clade-level LTR-RT classifications. Moreover, a recent update of REXdb [4] also provides classifications for other TEs, such as long interspersed nuclear repeats (LINEs), terminal inverted repeats (TIRs), and Helitrons (http://repeatexplorer.org/?page_id=918). We employed previous classifications of conserved protein domains to develop an automated, easy-to-use, and accurate classifier, named TEsorter. This software can be used to perform superfamily-level classification of TEs and to further classify LTR-RTs into clades. The Python code is freely available at https://github.com/zhangrengang/TEsorter.

The TEsorter package is implemented in Python3 and supports multiprocessing to reduce runtime. The conda approach is also supported to enable easier installation and better integration with other workflows. TEsorter was implemented using HMM profiles obtained from the TE protein domain databases GyDB (http://gydb.org) and REXdb [4]. For REXdb, multiple sequence alignments of domains of each clade were performed using MAFFT (https://mafft.cbrc.jp/alignment/software/) and HMM profiles were generated with HMMBuild [5].

To classify TE sequences, they are first translated in all six frames and the translated sequences are then searched against one of the two databases using HMMScan [5]. Hits with coverage <20% or E-value >1e-3 are discarded. For domains with multiple hits, only the best hit with the highest score is retained (Figure 1a). The classifications of TE superfamilies (e.g. LTR/*Copia*, LTR/*Gyspy*) and clades (e.g. *Reina* and *CRM* of *Gypsy*) are based directly on hits. For the *Copia* and *Gyspy* superfamilies, complete elements are identified based on the presence and order of conserved domains, including capsid protein (GAG), aspartic proteinase (AP), integrase (INT), reverse transcriptase (RT), and RNase H (RH), as described in Wicker *et al*. [6].

**Figure 1:**
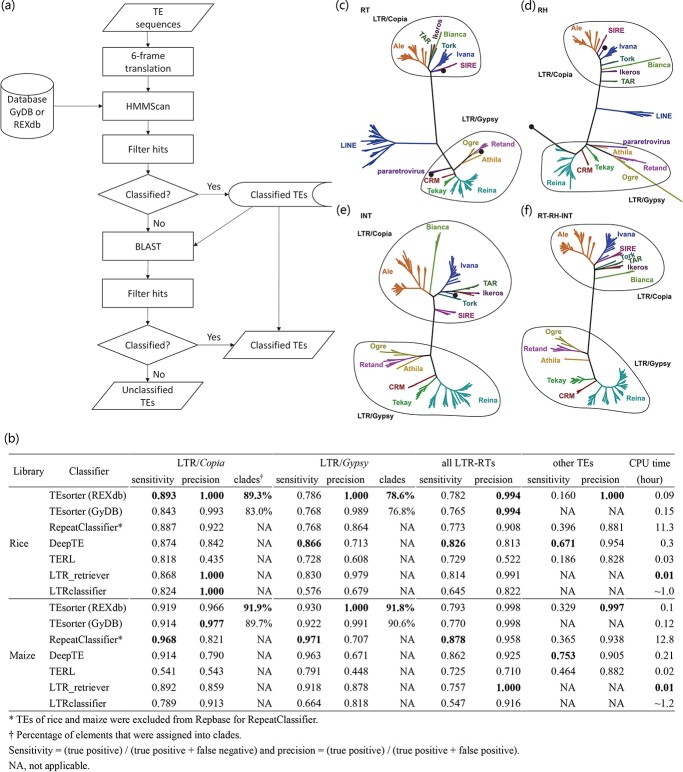
(a) A flowchart illustrating the TEsorter pipeline. (b) Comparison of performance among six TE classifiers, including TEsorter. (c-f) High consistency between classifications of TEs assigned by TEsorter and predicted phylogenetic relationships between TEs based on the RT (99.06%, c), RH (99.29%, d), INT (99.62%, e), and concatenated RT–RH–INT (100%, f) domains in rice. Conflicting results are highlighted with black circles. For detailed information, see https://github.com/zhangrengang/TEsorter/tree/master/example_data. Branches are colored based on TEsorter classifications.

Mutations such as frameshifts and domain losses may interfere with HMM-based classifications. To improve classification sensitivity, a two-pass strategy was implemented to classify non-autonomous TEs based on their sequence-level similarity to autonomous TEs (Figure 1a). The unclassified TE sequences are searched against the HMM-classified sequences using BLAST and then classified with the 80–80–80 rule (≥ 80 bp of alignment, ≥ 80% of sequence identity, and ≥ 80% of sequence coverage) [6]. To account for alignment uncertainties, this step is only utilized to classify sequences at the superfamily level.

To benchmark the classification performance of TEsorter, we first selected two curated TE libraries from rice (https://github.com/oushujun/LTR_retriever) and maize [7] with 2, 431 and 1, 546 sequence elements, respectively. We then compared TEsorter with five TE classifiers, including the RepeatClassifier module of RepeatModeler, the machine-learning-based classifiers DeepTE (https://github.com/LiLabAtVT/DeepTE) and TERL (https://github.com/muriloHoracio/TERL), the annotate_TE module of LTR_retriever (https://github.com/oushujun/LTR_retrieve), and the online-only LTRclassifier (http://LTRclassifier.ird.fr/). Some software was excluded from this analysis due to difficulty in making direct comparisons between it and TEsorter. For example, Inpactor (https://github.com/simonorozcoarias/Inpactor) requires LTR structural features and does not support sequences as sole input. TEclass (http://www.compgen.uni-muenster.de/teclass), REPCLASS [8], and PASTEC [9] only provide confident classifications at the order level, preventing comparison with the six other classifiers.

Another advantage of TEsorter over the aforementioned software is that it is capable of clade assignments to LTR-RT Copia or Gypsy elements. However, there is currently no available reference (or TE library) to enable evaluation of the clade-level assignments made by TEsorter. We therefore performed phylogenetic analyses based on the hypothesis that LTR-RT elements that were classified as being in the same clade are likely to have closer phylogenetic relationships. Briefly, protein domain sequences were extracted using TEsorter and aligned with MAFFT (https://mafft.cbrc.jp/alignment/software/). Phylogenetic trees were then reconstructed using the IQ-TREE JTT matrix distance model with bootstrap values of ≥50% after 1000 replicates [10]. To further validate the phylogenetic relationships of the clades assigned by TEsorter, we selected the same domains (i.e. RT, RH, INT, and concatenated RT–RH–INT) that were used by Neumann *et al*. [4] to determine phylogenetic relationships among clades of the LTR-RT *Copia* and *Gypsy* superfamilies.

This analysis demonstrated that TEsorter has the highest precision for classifying LTR-RTs when compared with the other TE classifiers tested (Figure 1b). When classifying the LTR-RT *Copia* and *Gyspy* superfamilies, the precision values of TEsorter with REXdb were both 1 in rice, and 0.966 and 1 in maize (Figure 1b). LTR_retriever and LTRclassifier had the same precision when classifying LTR-RT *Copia* in rice, however, their precision dropped significantly in maize and was also lower for the identification of LTR-RT *Gyspy* in rice (Figure 1b). We also tested the ability of these six pieces of software to classify TEs other than LTR-RTs. TEsorter with REXdb also had the highest precision in this test, with values of 1 in rice and 0.997 in maize (Figure 1b).

Unlike precision values, which were consistently higher in TEsorter, sensitivity varied among the tested TE classifiers. Specifically, TEsorter with REXdb and DeepTE had the highest value of sensitivity in classifying LTR-RT *Copia* and LTR-RT *Gyspy* in rice, respectively. RepeatClassifier had the highest sensitivity in classifying both LTR-RT *Copia* and LTR-RT *Gyspy* in maize (Figure 1b). Some TEs have become non-autonomous and could not be classified into superfamilies due to the loss of their characteristic protein domains, which was confirmed by searching against the Pfam database (http://pfam.xfam.org).

TEsorter had far shorter execution times than all other software tested. RepeatClassifier took more than 10 hours to finish its calculation, while TEsorter needed less than 10 minutes for the same calculation. Furthermore, Tesorter performed better with REXdb than with GyDB in most cases (Figure 1b), due to the systematic collection of plant LTR-RTs by Neumann *et al*. [4]. Overall, these results suggested that TEsorter is a well-rounded and competitive classifier at the superfamily level.

In addition to classification at superfamily levels, TEsorter was able to assign 76.8–91.9% of LTR-RT *Copia* or *Gypsy* elements into diverse clades in plants (Figure 1b). Moreover, the clade-level classification of TEsorter was found to be highly consistent with the reconstructed phylogeny, ranging from 99.06% based on the RT domain to 100% based on the concatenated RT–RH–INT domains (Fig. 1c-fa). Furthermore, phylogenetic relationships among the clades detected by TEsorter were in agreement with the clade classification of LTR-RT elements proposed by Neumann *et al*. [4]. These results revealed that TEsorter classifies with high confidence at the clade level and suggested that it is able to accurately assess the diversity of and phylogenetic relationships within the classified LTR-RTs.

Taken together, these results demonstrate that TEsorter has substantial improvements over the current tools in terms of both precision and execution time. Moreover, it is able to generate high-confidence classifications of LTR-RTs at the clade level. Overall, this software represents a significant step forward in TE classification.

## Data Availability

The Python script is freely available at https://github.com/zhangrengang/TEsorter.
